# Characterization of protein cargo of *Echinococcus granulosus* extracellular vesicles in drug response and its influence on immune response

**DOI:** 10.1186/s13071-023-05854-6

**Published:** 2023-07-29

**Authors:** María Celeste Nicolao, Christian Rodriguez Rodrigues, Magalí B. Coccimiglio, Camila Ledo, Guillermo H. Docena, Andrea C. Cumino

**Affiliations:** 1 Laboratorio de Zoonosis Parasitarias, IIPROSAM, Universidad Nacional de Mar del Plata (UNMdP), Funes 3350, Nivel Cero, 7600 Mar del Plata, Argentina; 2 Consejo Nacional de Investigaciones Científicas y Técnicas (CONICET), Buenos Aires, Argentina; 3 Departamento de Química, Facultad de Ciencias Exactas y Naturales, Universidad Nacional de Mar del Plata (UNMdP), Funes 3350, Nivel 2, 7600 Mar del Plata, Argentina; 4 Instituto de Estudios Inmunológicos y Fisiopatológicos (IIFP), La Plata, Argentina

**Keywords:** *Echinococcus granulosus*, Small extracellular vesicles, Immunomodulation, Parasite–host interaction, Dendritic cells, Metformin, Albendazole

## Abstract

**Background:**

The *Echinococcus granulosus *sensu lato species complex causes cystic echinococcosis, a zoonotic disease of medical importance. Parasite-derived small extracellular vesicles (sEVs) are involved in the interaction with hosts intervening in signal transduction related to parasite proliferation and disease pathogenesis. Although the characteristics of sEVs from *E. granulosus* protoscoleces and their interaction with host dendritic cells (DCs) have been described, the effect of sEVs recovered during parasite pharmacological treatment on the immune response remains unexplored.

**Methods:**

Here, we isolated and characterized sEVs from control and drug-treated protoscoleces by ultracentrifugation, transmission electron microscopy, dynamic light scattering, and proteomic analysis. In addition, we evaluated the cytokine response profile induced in murine bone marrow-derived dendritic cells (BMDCs) by qPCR.

**Results:**

The isolated sEVs, with conventional size between 50 and 200 nm, regardless of drug treatment, showed more than 500 cargo proteins and, importantly, 20 known antigens and 70 potential antigenic proteins, and several integral-transmembrane and soluble proteins mainly associated with signal transduction, immunomodulation, scaffolding factors, extracellular matrix-anchoring, and lipid transport. The identity and abundance of proteins in the sEV-cargo from metformin- and albendazole sulfoxide (ABZSO)-treated parasites were determined by proteomic analysis, detecting 107 and eight exclusive proteins, respectively, which include proteins related to the mechanisms of drug action. We also determined that the interaction of murine BMDCs with sEVs derived from control parasites and those treated with ABZSO and metformin increased the expression of pro-inflammatory cytokines such as IL-12 compared to control cells. Additionally, protoscolex-derived vesicles from metformin treatments induced the production of IL-6, TNF-α, and IL-10. However, the expression of IL-23 and TGF-β was downregulated.

**Conclusions:**

We demonstrated that sEV-cargo derived from drug-treated *E. granulosus* protoscoleces have immunomodulatory functions, as they enhance DC activation towards a type 1 pro-inflammatory profile against the parasite, and therefore support the proposal of a new approach for the prevention and treatment of secondary echinococcosis.

**Graphical abstract:**

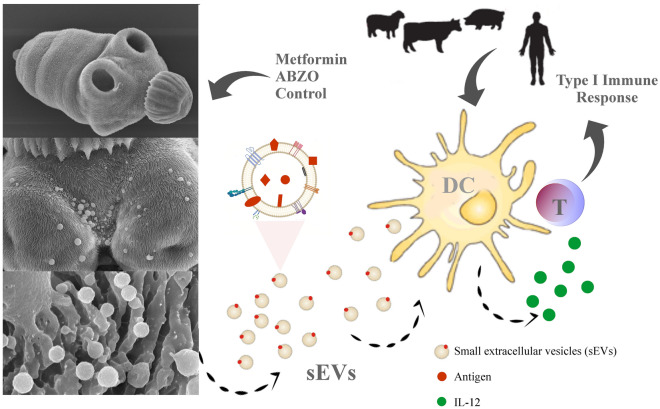

**Supplementary Information:**

The online version contains supplementary material available at 10.1186/s13071-023-05854-6.

## Background

Parasite helminth cells, like almost all cells, release extracellular vesicles (EVs), which comprise phospholipid bilayer-enclosed vesicles that carry parasite components including proteins, lipids, glycans, and nucleic acid [1]. Traditionally, EVs are classified into exosomes and ectosomes or microvesicles based on their size, composition, and intracellular site of origin. Exosomes are considered small vesicles of typically 30–200 nm which originate from the inward budding of late endosomes, while ectosomes are plasma membrane-derived vesicles of a wider size range (usually 100–1000 nm) [2, 3]. Since commonly isolated EVs are heterogeneous populations with diverse origins, we will employ the term “small extracellular vesicles” (sEVs) to refer to EVs of less than 200 nm [3, 4]. These sEVs have been shown to enable communication not only between parasites, but also in parasite–host interactions [5]. The parasite–host interplay is essential for the success of parasite infection, colonization, and development, processes in which the parasite generally passes through different life stages or forms that involve morphological and antigenic changes, and consequently the host immune system modulation [6]. The manipulation of the host response is particularly important in long-term infections, such as helminthiases, as it promotes parasite survival [7].

Cystic echinococcosis (CE) is a chronic helminthic zoonotic disease caused by the larval stage of *Echinococcus granulosus *sensu lato that affects more than one million people worldwide [8]. In humans, the parasite develops as slow-growing cysts or metacestodes containing hydatid fluid and protoscoleces that mostly settle in the liver and lungs [9]. In recent years, an increasing number of reports on the modulatory role of excretory/secretory (E/S) products released by *Echinococcus* spp. have been published [10–14]. Recent studies revealed that the E/S products of helminths contain sEVs which may be involved in the interaction with the host and could have immunomodulatory functions [1]. For example, the EVs secreted by *Echinococcus multilocularis* can be internalized by murine macrophages and have regulatory effects including suppression of pro-inflammatory cytokines and nitric oxide production and induction of key components of the lipopolysaccharide (LPS)-TLR4 pathway [15]. In *E. granulosus*, we demonstrated that the sEVs obtained from in vitro cultures of protoscoleces carry several immunoregulatory proteins and interact with bone marrow-derived dendritic cells (BMDCs) inducing an unconventional activation profile [16]. It was also reported that EVs obtained from hydatid fluid and protoscoleces of patients with CE can immunomodulate murine peripheral blood mononuclear cells and, consequently, inhibit CD4^+^ and CD8^+^ T-cell proliferation and the release of pro-inflammatory cytokines [17]. In addition, sEVs from this parasite have been shown to possess a higher rate of internalization by liver cells and greater reactivity with anti-echinococcosis positive serum with respect to larger EVs, suggesting that they play a more relevant role in the parasite–host interaction during *E. granulosus* infection [18].

A major risk associated with CE is the possibility of cyst rupture, which causes the release of large amounts of hydatid fluid and protoscoleces, resulting in a secondary hydatid infection and a high rate of free sEVs [16, 19]. Furthermore, in our previous report, we demonstrated that intact murine *E. granulosus* metacestodes can release sEVs into the culture medium, and we hypothesized that they probably cross the parasite laminated layer through the binding of their cargo proteins to the calcium inositol hexakisphosphate deposits [16]. Likewise, plasma exosomes from CE patients were found to contain parasite proteins, indicating that *E. granulosus*-derived exosomes could leak and end up in the circulation, and thus could be involved in vesicle–vesicle and vesicle–cell interaction [20, 21]. The hydatid cyst turgidity can be altered by the use of several anthelminthic drugs [22, 23]. The loss of turgidity is associated with an increase in cyst permeability and probably with a higher release of hydatid fluid and consequently the intracystic sEVs. Additionally, it is known that during CE and alveolar echinococcosis, pharmacological treatment with albendazole influences T helper 1 (Th1)/Th2 cytokine production, altering the immune response towards a Th1-shifted response [24–27]. Based on all of these factors, and because pharmacological treatment can modify the sEV cargo in other cellular systems [28–30], the aim of this work was to characterize the protein cargo of sEVs from *E. granulosus* protoscoleces and the immune response profile of dendritic cells (DCs) that they induce after treatment with drugs such as albendazole sulfoxide (ABZSO, the main metabolite of albendazole, the current drug of choice for echinococcosis) and metformin (an experimental drug with reported effects against both CE and alveolar echinococcosis) [31, 32].

## Methods

### Ethics statement

The animal study was performed in accordance with the guidelines of the National Health Service and Food Quality (SENASA), Argentina. The experimental protocols were evaluated and approved by the Animal Experimental Committee at the Faculty of Exact and Natural Sciences, Mar del Plata (permit number: RD544-2020; RD624-625-2021).

### Experimental animals

Healthy female CF-1 mice (28–35 g) were supplied by SENASA and housed in conventional facilities in the bioterium of the National University of Mar del Plata. Each experiment was performed using a minimum number of animals. The animals (five mice per cage) were monitored daily and kept under controlled laboratory conditions (temperature ± 20 °C, 12 h light/12 h dark with lights off at 8.00 p.m.). Water and food were administered ad libitum, and cages were cleaned and filled with fresh sawdust every 3–4 days. The mice received ketamine/xylazine (50 mg/kg/mouse/5 mg/kg/mouse) as an anesthetic and were sacrificed by cervical dislocation. All attempts were made to reduce suffering.

### In vitro culture of protoscoleces

Protoscoleces of *E. granulosus* were isolated from the lungs and livers of infected cattle slaughtered at the abattoir in the province of Buenos Aires, Argentina. Hydatid cysts were aseptically opened to recover the protoscoleces, which were thoroughly washed in phosphate-buffered saline (PBS). Then, for each treatment, a total of 9000 protoscoleces were cultured in Leighton tubes in Medium 199 (Gibco) with antibiotics (penicillin, streptomycin, and gentamicin 100 μg/ml), glucose (4 mg/ml), and with or without the addition of anti-echinococcal drugs, and maintained at 37 °C without changing the medium for 7 days. In vitro protoscolex sublethal pharmacological treatments were assayed with 10 mM metformin (1,1-dimethylbiguanide hydrochloride, Sigma-Aldrich, USA) and 5 μg/ml (equivalent to 17.8 μM) ABZSO (kindly provided by C. Salomon, National University of Rosario, Argentina), which were dissolved in water and dimethyl sulfoxide, respectively [31]. Corresponding controls were incubated with dimethyl sulfoxide. Vitality was assessed before and after pharmacological treatments using the methylene blue exclusion test, which remained above 90% after all assays as described previously [33].

### Extracellular vesicle purification

Extracellular vesicles were obtained from three independent experiments by differential centrifugation [34]. Briefly, the protoscolex culture medium was centrifuged at 300×*g* for 10 min, then at 2000×*g* for 10 min, and finally at 10,000×*g* for 30 min. The resulting supernatant was ultracentrifuged at 100,000×*g* for 1 h in an Optima LE-80K ultracentrifuge (Beckman) using a 90 Ti rotor. These isolated EVs from pellets were washed with 3 ml of PBS and centrifuged at the same high speed for 30 min to remove contaminating proteins [35]. Finally, the EVs were resuspended in 30 µl PBS and stored at −80 °C until use. Protein concentration was determined using 1 μl of the sample by measuring absorbance at 280 nm with a NanoDrop ND-1000 spectrophotometer. The sEVs were free of endotoxins, as determined by the *Limulus* amebocyte lysate (LAL) method.

### Dynamic light scattering (DLS)

The size distribution profile of EVs obtained from protoscoleces of *E. granulosus* was determined by DLS using a Zetasizer Nano (Nano ZS ZEN3600, Malvern Panalytical, Malvern, UK) at the Instituto de Investigaciones Fisicoquímicas Teóricas y Aplicadas (INIFTA, Argentina), as described previously [16].

### Transmission electron microscopy (TEM)

For TEM, 4 µl of EVs were fixed in 2% paraformaldehyde in PBS and sent to the Centro Regional de Investigaciones Básicas y Aplicadas de Bahía Blanca (CRIBABB), Argentina. Samples were processed by an external service as described previously [16, 34]. Images were obtained using a JEOL JSM 100CX II TEM instrument.

### Proteomic analysis

EVs (10 μl with ≈ 60 μg total protein) were electrophoresed into the resolving gel of a 10% sodium dodecyl sulfate–polyacrylamide gel (SDS-PAGE) for 1 cm. The samples were then stained with colloidal Coomassie Blue G-250, cut from the gel, and subjected to mass spectrometry analysis and protein identification by an external service (CEQUIBIEM proteomic service, Buenos Aires, Argentina). All proteomic analyses were performed as described previously [16]. Samples were processed using nano-HPLC [high-performance liquid chromatography] (EASY-Spray Accucore, Thermo Scientific, West Palm Beach, FL, USA) coupled to a mass spectrometer with Orbitrap technology (Q Exactive, Thermo Scientific, West Palm Beach, FL, USA). The resulting data were analyzed using Proteome Discoverer software version 1.4 (Thermo Scientific). Further analyses were performed using proteins with at least two peptides in duplicate.

### Computational predictive methods based on sequence data

In silico analyses to functionally classify peptides and identify parasite proteins were performed using the UniProt and Reactome pathway databases and manually performed based on the literature. The proteins previously classified as “antigens” were further analyzed to identify linear B-cell epitopes using BepiPred 2.0 based on the hidden Markov model and propensity scoring method (with a threshold value of 0.6). Additionally, the prediction of *N*- and *O*-glycosylation sites in these proteins was performed with NetNGlyc v1.0 and NetOGlyc v4.0 web servers (using cutoff values of > 0.5 and > 0.75, respectively). Conserved domains and a family of immunoregulatory proteins were assigned using the Conserved Domains Database v3.15 and CDART (Conserved Domain Architecture Retrieval Tool), and submitted to the SWISS-MODEL server to select within all known experimental 3D structures in the database, a possible template combining properties from the target–template alignment and the template structure. Furthermore, the “uncharacterized, hypothetical, conserved, or expressed proteins” were also analyzed for epitope prediction using the same parameters as in “antigens.” The probable position and number of transmembrane regions of these sequences were predicted using the HMMTOP, TMHMM, and DAS web servers. Finally, to analyze classical or nonclassical secretory pathways, we used the SignalP and SecretomeP servers [36], respectively.

### Generation, culture, and activation of BMDCs

Murine-differentiated DCs were obtained from the bone marrow of femurs and tibias of CF-1 mice (6–8 weeks old) as described previously [37]. Briefly, cell suspensions were depleted of red blood cells using NH_4_Cl buffer. Cells were cultured in complete RPMI 1640 medium in the presence of 100 ng/ml Flt3-L (R&D Systems), as described previously [16]. Finally, the primary cell cultures were characterized by fluorescence-activated cell sorting (FACS) using fluorescence-conjugated monoclonal antibodies directed against CD11b (M1/70), CD11c (HL3), CD3 (145-2C11), CD45R/B220 (RA3-6B2), Siglec-H (eBio440c), CD172a (P84), and CD24 (M1/69) (eBioscience, San Diego, CA, USA). Approximately 70–90% of the cells were CD11c^+^.

BMDCs (1 × 10^6^ per milliliter) were washed, pelleted, and cultured in the absence or presence of 10 µl EVs (60 μg total protein in relation 1:1) from control or 10 mM metformin- or 5 μg/ml ABZSO-treated samples for 30 min at 37 °C. Cells were then gently resuspended in complete RPMI 1640 medium and maintained for 18 h before being harvested for subsequent gene expression studies. In addition, a group of cells were stimulated for 18 h with 100 ng/ml of LPS (Sigma-Aldrich) as a positive control of maturation.

### Quantitative reverse transcription polymerase chain reaction (qRT-PCR)

Total RNA was extracted from BMDCs using TRIzol^®^ reagent (Thermo Fisher Scientific) according to the manufacturer’s instructions. The quantification and purity of nucleic acids were evaluated using a NanoDrop ND-1000 spectrophotometer. Total RNA (300 ng) was reverse transcribed using Oligo-dT and M-MLV RT (Thermo Fisher Scientific). The sequences of the specific primers used were as follows: interleukin 10 (IL-10) (F: CCAAGCCTTATCGGAAATGA; R: TTTTCACAGGGGAGAAATCG), transforming growth factor beta (TGF-β) (F: TTGCTTCAGCTCCACAGAGA; R: TGGTTGTAGAGGGCAAGGAC), IL-6 (F: AGTTGCCTTCTTGGGACTGA; R: TCCACGATTTCCCAGAGAAC), tumor necrosis factor alpha (TNF-α) (F: AGCCCCCAGTCTGTATCCTT; R: CTCCCTTTGCAGAACTCAGG), indoleamine-pyrrole 2,3-dioxygenase (IDO) (F: GGCTAGAAATCTGCCTGTGC; R: AGAGCTCGCAGTAGGGAACA), IL-12p35 (F: CATCGATGAGCTGATGCAGT; R: CAGATAGCCCATCACCCTGT), IL-23p19 (F: GACTCAGCCAACTCCTCCAG; R: GGCACTAAGGGCTCAGTCAG). Gene expression analysis was performed using the SYBR^®^ Green PCR Master Mix (Applied Biosystems) for the detection of PCR products on an Applied Biosystems StepOne cycler. The PCR conditions were as follows: a holding stage of 95 °C (10 min), 40 cycles steps of 95 °C (15 s), 60 °C (1 min), and a melting curve stage of 95 °C (15 s), 60 °C (1 min), and 95 °C (15 s). The expression levels were assessed using the 2^−ΔΔCt^ method, and each experiment was performed in triplicate with appropriate non-template controls. The relative abundance of each transcript was determined by normalizing it to the reference *GAPDH* gene.

### Statistics

The statistical analysis of the experimental trials was performed using the one-way analysis of variance (ANOVA) test followed by Tukey's post hoc analysis. All data are shown as arithmetic mean ± standard deviation, and the *P*-values are specified in each graph.

## Results

### *Echinococcus granulosus* protoscoleces release sEVs with similar proteomic characteristics under control conditions and metformin or ABZSO treatment

**Fig. 1:**
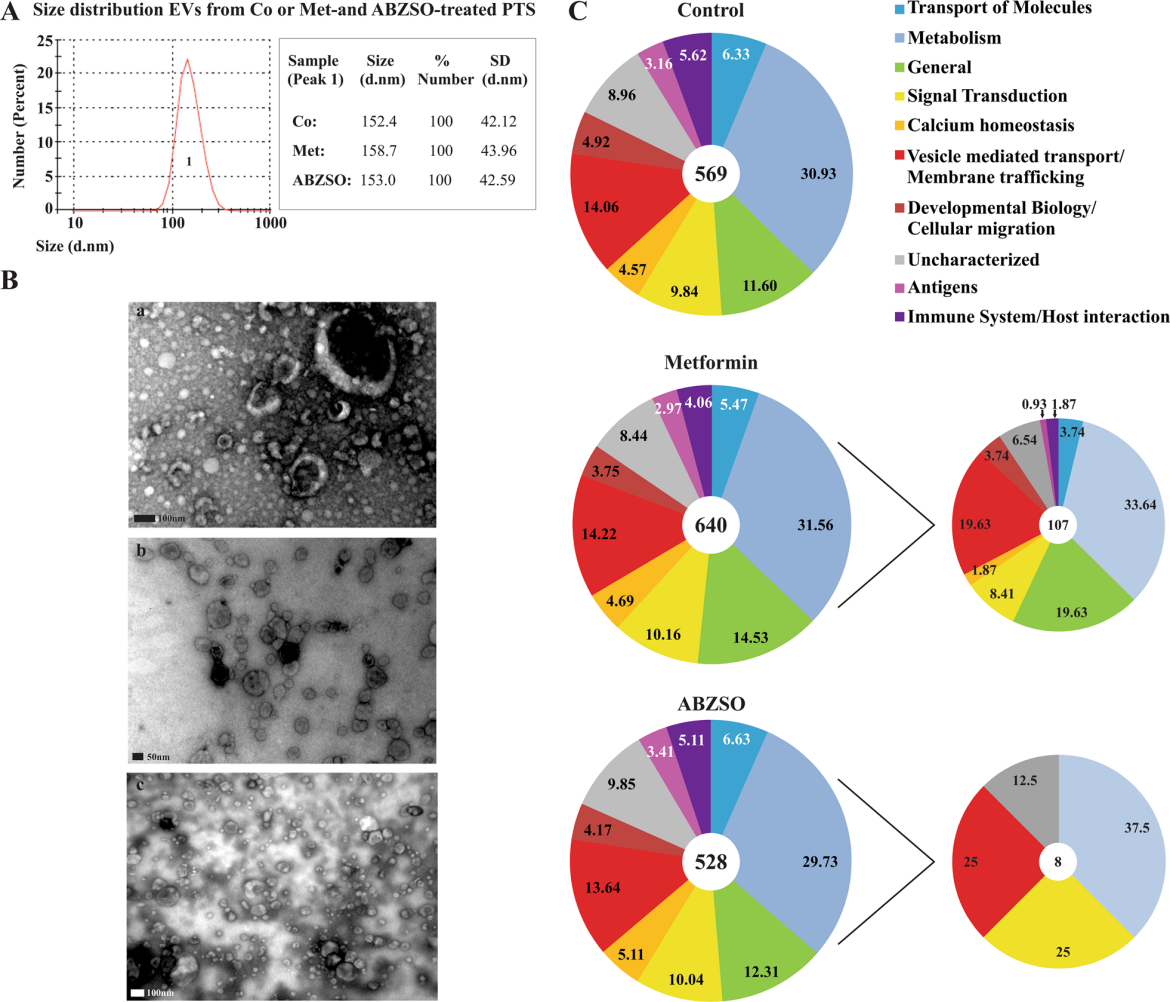
Characterization of extracellular vesicles obtained from *E. granulosus* protoscoleces during metformin and ABZSO treatment. **A** Size distribution curves determined by DLS of isolated EVs from control (Co) and metformin (Met)- or albendazole sulfoxide (ABZSO)-treated protoscoleces. The DLS histogram corresponds to the control condition. **B** Morphological characterization of control EVs by TEM revealed abundant vesicles with a diameter between 100 and 150 nm and morphology similar to exosomes. The scale indicates 100 nm in a (Co) and c (ABZSO) and 50 nm in b (Met). **C** Functional classification and proportion of expression of proteins identified from EVs isolated from control and metformin- or ABZSO-treated protoscoleces (main graphics). Right panel corresponds to proteins expressed exclusively in EVs from metformin- or ABZSO-treated protoscoleces

To analyze the effects of metformin and ABZSO on *E. granulosus* protoscolex EV production and the protein cargo identity, EVs were purified by differential centrifugation, and the expression of CD9 was determined by western blot as described previously [16]. The size distribution of isolated EVs was assessed using DLS (Fig. 1A), which showed a mean diameter of 150 nm. Additionally, the TEM analysis confirmed the presence of cup-shaped structures of 50–150 nm (Fig. 1B,) consistent with exosome-like vesicles and in accordance with the DLS outcome.

Proteomic analysis of EVs obtained from control and metformin- or ABZSO-treated protoscoleces revealed the presence of 569, 640, and 528 proteins, respectively (Additional file 1: Tables S1–S3). Proteins were classified into 10 categories based on analysis using the Reactome database and information from the literature. Despite the differences in the total protein amount, the proportion of proteins in each functional category remained constant in all samples (Fig. 1C and Additional file 1: Tables S1–S3). Notwithstanding the high overlap of proteins detected in these EVs, some of them were exclusively identified in EVs derived from metformin-treated parasites such as guanosine monophosphate (GMP)-synthase (W6UEZ1), estradiol 17β-dehydrogenase (W6U9Y5), and 5′-AMP-activated protein kinase subunit γ (W6V734), among others (Fig. 1C and Additional file 1: Table S2). We also analyzed the possible changes in EV-protein cargo linked to the mechanism of action of ABZSO and metformin with respect to the control. In EVs purified from metformin-treated parasites, we found several overrepresented enzymes involved in reactive oxygen species (ROS) scavenging activity, energy and metabolic processes (such as glycolysis, tricarboxylic acid cycle, and nucleotide biosynthesis), and lysosomal and mitochondrial activity; whereas the basement membrane proteins were less enriched with this drug. On the other hand, in EVs purified from ABZSO-treated protoscoleces, we detected cytoskeletal proteins such as tubulins and carbon metabolism enzymes with downregulated expression, accompanied by upregulation of 26S proteasome components and heat shock proteins (Additional file 2: Table S4).

### *Echinococcus granulosus* sEVs are an antigen source independent of drug treatment

**Fig. 2:**
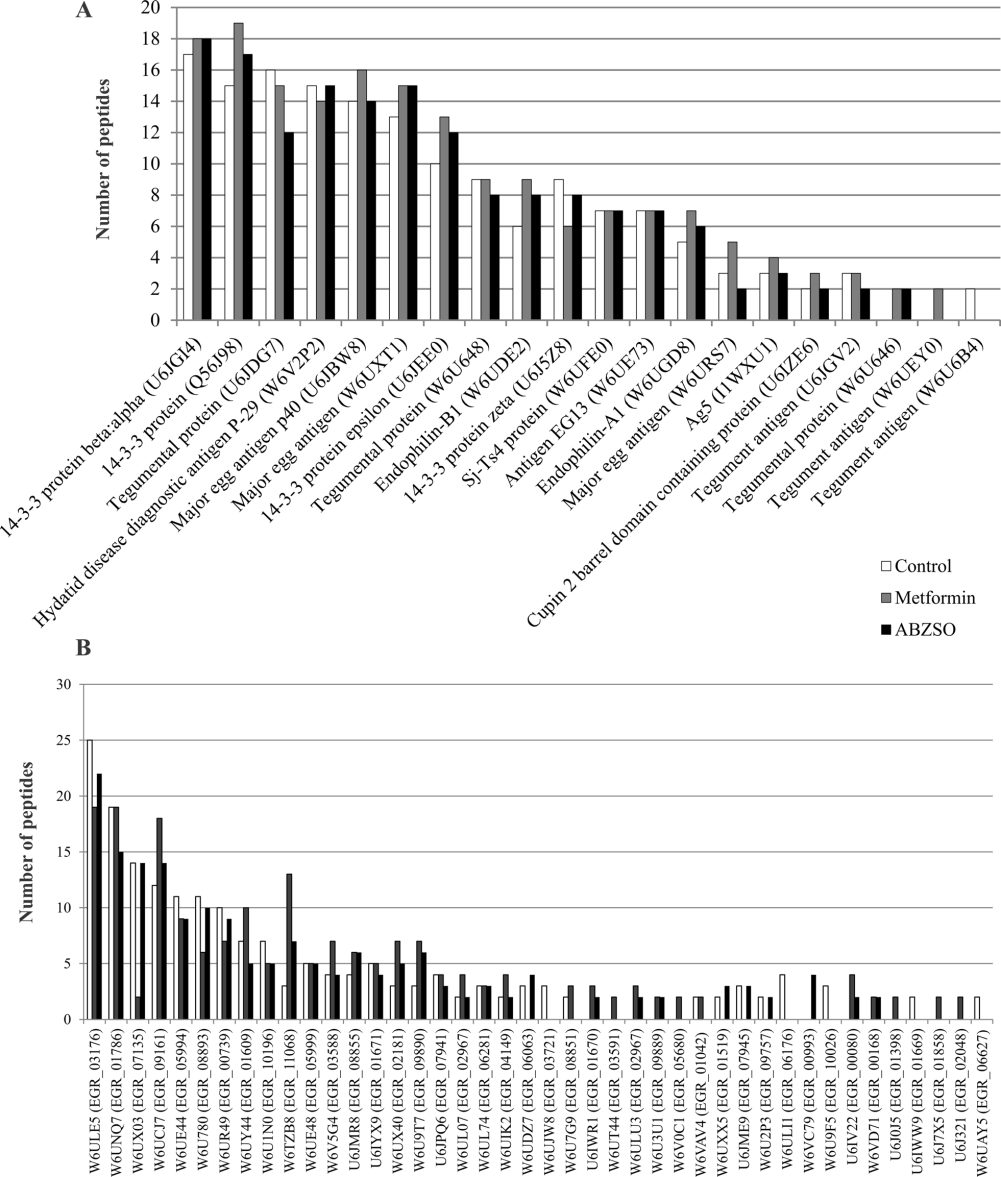
Antigenic cargo of sEVs from *E. granulosus* protoscoleces determined by proteomic analysis. **A** Proteins expressed in sEVs from control (white bars) and metformin-treated (gray bars) or ABZSO-treated (black bars) parasites, which were previously characterized as *Echinococcus*-specific antigens. **B** Putative antigens expressed in sEVs obtained from the samples indicated in A. Putative antigen identification was realized through the analysis of uncharacterized proteins shown in Additional file 4: Table S6 with the epitope predictor software BepiPred 2.0

Based on the proteomic data, we identified at least 20 known *Echinococcus*-specific antigens with molecular weight lower than 700 kDa. Most of them were represented in equal proportions and identities in the different samples, including sEVs obtained from control conditions and under pharmacological treatment (Fig. 2A and Additional file 3: Table S5). Only two tegumental antigens (W6U646 and W6UEY0) were detected in sEVs from drug-treated parasites and were absent in control samples. In addition, antigen EG13 (W6UE73), major egg antigen (W6URS7), tegumental antigen (W6U646), and Ag5 (I1WXU1) were the antigens with the highest number of predicted epitopes and glycosylation sites (Additional file 3: Table S5). Except for the 14-3-3 proteins, all antigens showed the presence of at least one putative glycosylation site.

Furthermore, to gain insight into the putative role of the uncharacterized proteins in the parasite–host interaction, we analyzed 70 uncharacterized proteins using epitope predictor software, which allowed the identification of 42 unknown putative antigens (Fig. 2B and Additional file 4: Table S6). Half of them could be secreted by nonclassical secretory pathways (Sec-P score > 0.6), and 26% (11/42) were predicted to contain at least one transmembrane domain (Additional file 4: Table S6). In particular, proteins such as W6UCJ7, W6UNQ7, and W6TZB8 are of great interest because they are highly expressed in sEVs and exhibit 31, 15, and 10 predicted epitopes, respectively. Thus, sEVs could transfer these antigens to regulate host cell functions, including the modulation of DCs and direct or indirect activation of T and B cells.

### Proteomic profiling of sEVs identifies putative immunomodulatory and host-interacting proteins

**Fig. 3:**
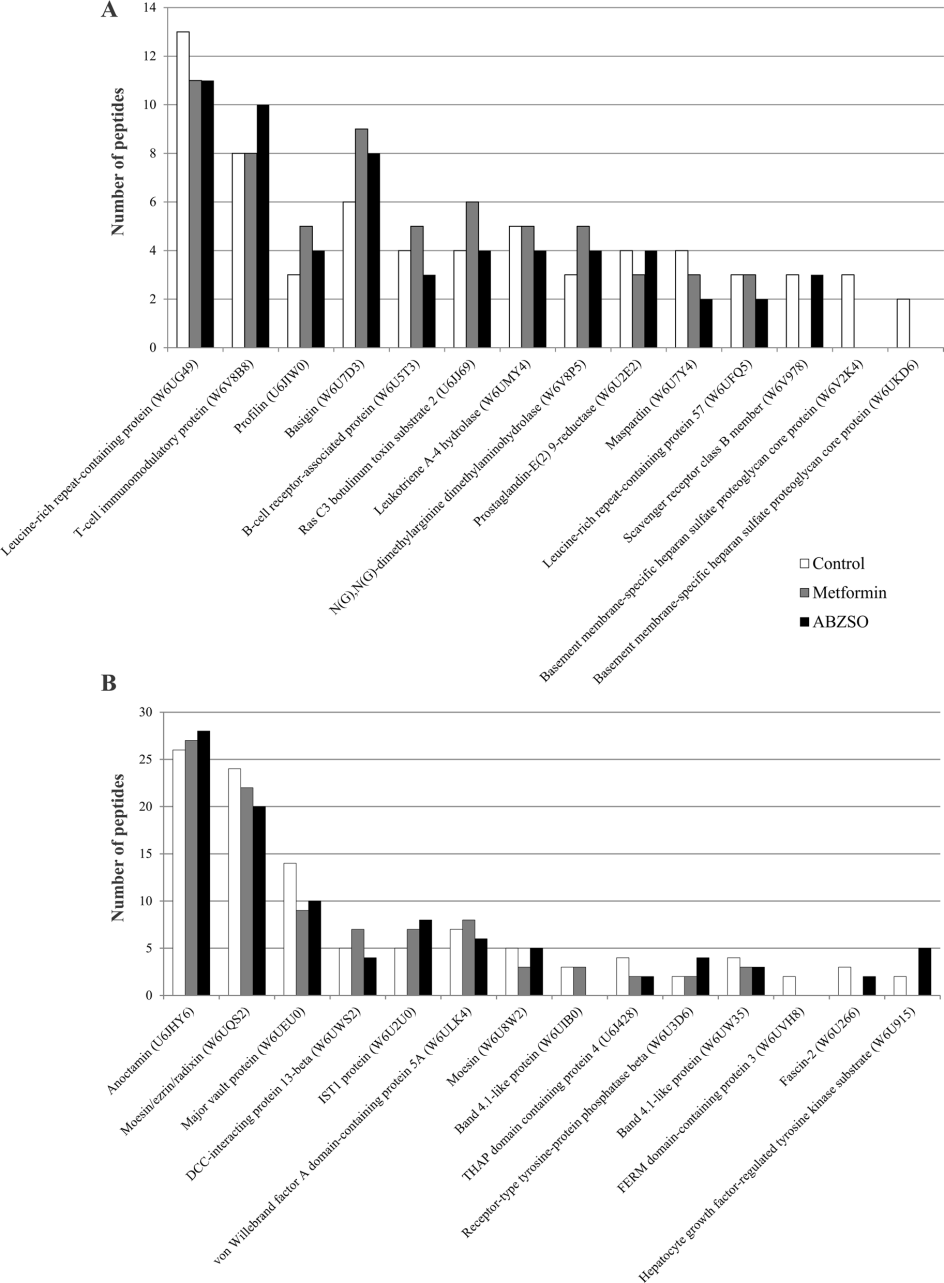
Host-immunomodulatory cargo of sEVs from *E. granulosus* protoscoleces determined by proteomic analysis. **A** Putative immune modulator and **B** host-interacting proteins expressed in sEVs from control (white bars) and metformin-treated (gray bars) or ABZSO-treated (black bars) parasites identified from the literature. Most of them were represented in similar proportions in the different parasite samples

To identify potential immune modulators and host-interacting proteins in the sEV protein cargo, we generated a summary of the most abundant candidates in each sample set (Fig. 3). In *E. granulosus* sEVs, 13 proteins encoded by genes previously associated with host immune defense were identified and expressed in the adult and larval forms (EG_0454, EG_4838, EG_03471, EG_03469, EG_03470, EG_03468, EG_02555, and EG_0838), and some of them were also expressed in oncospheres (EG_5022, EG_08217, EG_01663, EG_03926, EG_06615) [38].

Interestingly, we detected a T-cell immunomodulatory protein (TIP, W6V8B8 of 909 residues, Fig. 3A and Additional file 1: Fig. S1) as a main transmembrane protein in *E. granulosus* sEVs. The predicted Eg-TIP protein contains one N-terminal GTPase activator domain (first 260 residues with similarity to Rab-like small GTPases), two transmembrane domains (including 256–278 and 806–828 residues), a segment identified as a domain in adhesion like-VCBS (297–363 amino acids, Pfam13517 and cl21563 VCBS superfamily), and the characteristic region with structural identity with TIP orthologs in flatworms, protozoa, and vertebrates (Additional file 1: Fig. S1).

We also identified two small enzymes within the *E. granulosus* sEVs that are involved in the most distal steps of inflammatory eicosanoid synthesis. Prostaglandin-E_2_ 9-ketoreductase (Eg-PE2R, W6U2E2) and LTA4 hydrolase (Eg-LTA4H, W6UMY4) (Fig. 3A). The full-length open reading frames of Eg-PE2R and Eg-LTA4H identified by Basic Local Alignment Search Tool (BLAST) search predicted proteins of 304 and 639 amino acids, with 44.3% and 39.1% identity and quaternary structure similar to rabbit PE2R (Protein Data Bank [PDB] ID 1q5m.1A) and human LTA4H (PDB ID 6enc.1A) orthologs, respectively (Additional file 1: Fig. S1).

Structural analysis revealed two leucine-rich repeat proteins, W6UG49 and W6UFQ5, with 21% and 26% identity, respectively. Both proteins possess leucine-rich repeat (LRR)-containing domains responsible for sensing pathogen patterns as the first line of defense. Additionally, another transmembrane glycoprotein of 586 residues (W6V978) was recognized in sEVs from control and ABZSO-treated parasites, associated with the CD36 superfamily, which showed 28% identity with class B scavenger receptor. Finally, two modular proteins (W6V2K4 and W6UKD6 of 7853 and 846 residues, respectively) with identity to basement membrane-specific heparan sulfate proteoglycan core proteins were detected only in sEVs obtained from control protoscoleces. These proteins could combine the heparin-sulfate moiety allowing the binding and the mitogenic activity of fibroblast growth factor (Additional file 1: Fig. S1).

### Small EVs from treated *E. granulosus* modify cytokine expression in BMDCs

**Fig. 4:**
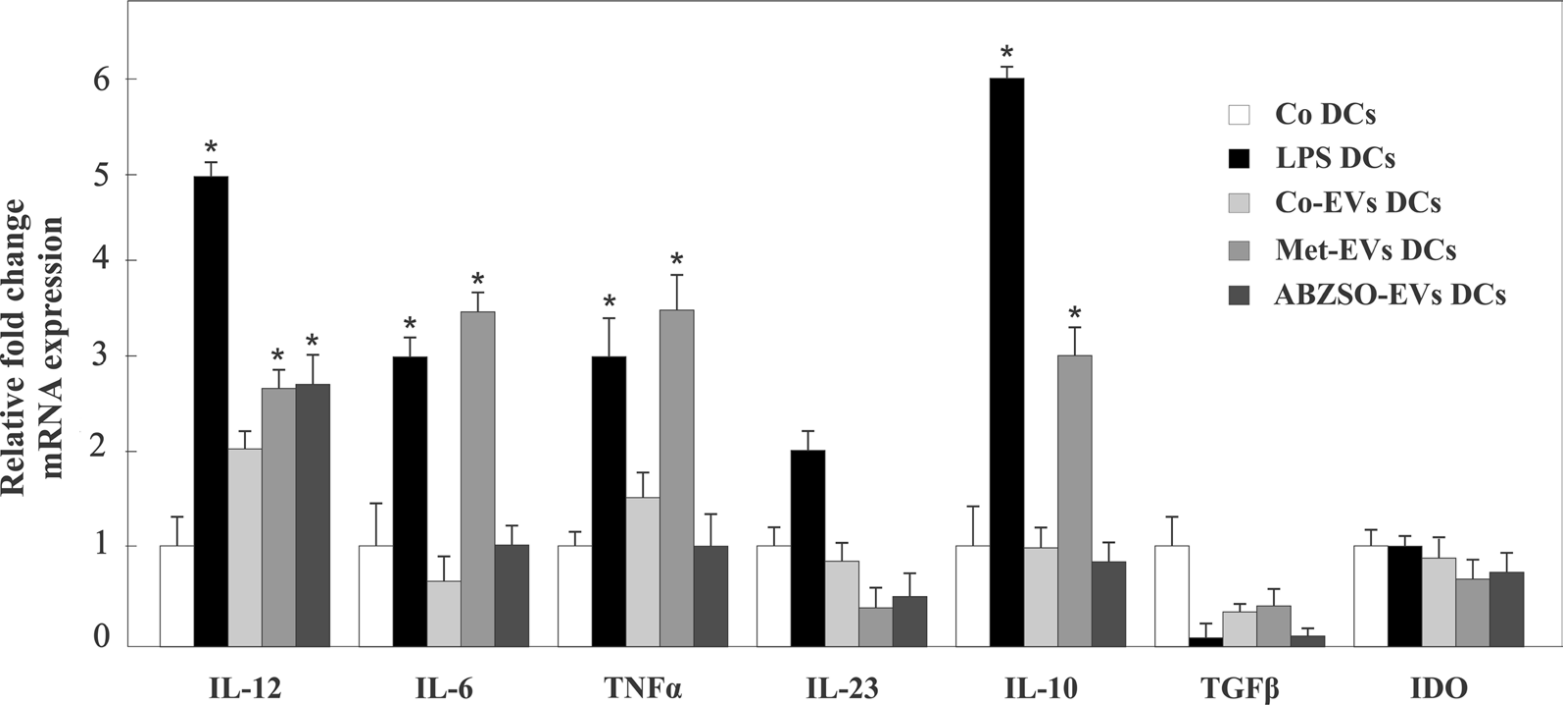
Cytokine gene expression in BMDCs after *E. granulosus* protoscolex sEV exposure. Quantitative PCR analysis of IL-12, IL-6, TNF-α, IL-23, IL-10, TGF-β, and IDO from total RNA of BMDCs incubated for 18 h under control conditions (Co DCs) or exposed to sEVs from control protoscoleces (Co-EV DCs) or protoscoleces treated with 10 mM metformin (Met-EV DCs) or 5 μg/ml ABZSO (ABZSO-EV DCs). BMDCs incubated with 100 ng/ml of LPS (LPS DCs) were used as positive control. Amplification of *GAPDH* was used as a reference gene. Fold change expression values are plotted. Data are the mean ± SD of three independent experiments. *Statistically significant difference (*P* < 0.05) compared with control

The interaction of sEVs with DCs leads to the production of cytokines and skewing of T-cell responses towards a pro-inflammatory (Th1: IL-12, TNF-α; Th17: IL-6, IL-23) or a tolerogenic profile (Treg: IL-10, TGF-β), the results of which are useful for understanding the type of parasite–host interplay mediated by sEVs. Thus, we next analyzed cytokine gene expression in DCs by qRT-PCR after 18 h of sEV exposure (Fig. 4). BMDCs cultured in the presence of sEVs isolated from *E. granulosus* demonstrated a tendency to increase the expression of pro-inflammatory cytokines such as IL-12 and TNF-α, and a trend to down-modulate TGF-β and IL-6 compared to control cells (Fig. 4). No differences were observed in the expression levels of IL-23, IL-10, or IDO, an inducible enzyme for tryptophan catabolism, which is a feature of tolerogenic DCs that have T-cell regulatory properties. Surprisingly, sEVs isolated from metformin-treated protoscoleces induced an increase in pro-inflammatory cytokines such as IL-6, TNF-α (2.5-fold change), and IL-12 (1.5-fold change) compared to untreated cells. It is also important to note the increase in IL-10 expression (twofold change compared to control cells), a cytokine involved in the development of different T-cell profiles (Treg and Th2). Conversely, IL-23, TGF-β, and IDO genes showed a nonsignificant decrease in expression compared to the control. Unlike what was observed with sEVs from metformin-treated parasites, the messenger RNA (mRNA) levels of pro-inflammatory cytokines expressed by BMDC that were exposed to sEVs from ABZSO-treated protoscoleces were similar to those in control cells, except for the significant upregulation of IL-12 (1.5-fold change).

## Discussion

Helminth-released EVs are considered to be one of the strategies employed to deliver signals to improve parasite development, growth, and survival through active communication with the host [1]. Previous reports have evidenced the properties of sEVs released by the larval stage of *E. granulosus* and their interaction with host cells such as hepatocytes, macrophages, and DCs [16, 18, 39]. In this work, we isolated and characterized sEVs from control and drug-treated *E. granulosus* protoscoleces and studied the immune response profile induced on murine DCs.

The production, release, and uptake of EVs are regulated by intracellular and extracellular stimuli [40]. Likewise, therapeutic interventions represent both types of stimuli, in which drugs can inhibit or activate the biogenesis and release of exosomes [41] and alter their morphological characteristics and cargo [42, 43]. Thus, in this context, we aimed to identify potential differences in the protein pattern and immune response of sEVs from *E. granulosus* protoscolex cultures under pharmacological treatment as a potential strategy for monitoring drug response. Consistently with several reports, the size of sEVs determined by DLS and corroborated by TEM was between 50 and 200 nm (Fig. 1A, B). This diameter was slightly larger than our previous determination for the exosome-like vesicles of the *E. granulosus* larval stage [16, 44]. Also, we have previously shown that an increase in intracellular calcium in the presence of loperamide was able to increase the formation and secretion of *E. granulosus* EVs, similarly to other reports, as well as a greater abundance in their protein cargo [16, 45, 46]. Here, our hypothesis was partially verified, since sEVs purified from all conditions showed a similar protein pattern (Fig. 1C). However, although in control samples and in the presence of ABZSO the total amount of proteins was similar, in the presence of metformin it was greater (Fig. 1C). This could be associated with an enhanced release of EVs under metformin treatment, as has been reported in mesenchymal stem cell-derived EVs [47]. It is known that metformin, through the activation of 5′ AMP-activated protein kinase (AMPK), induces indirect inhibition of target of rapamycin complex I (TORC1) in the parasite, and as has been described in animal cell models, sustained inhibition of TORC1 activates the exosome release concomitantly with activation of autophagy [48, 49].

In particular, we detected 107 and eight exclusive proteins in the sEV-cargo from metformin- and ABZSO-treated parasites, respectively, which are probably associated with drug-induced cellular alterations [31, 32, 48, 50]. In fact, we identified proteins that modified their abundance with respect to the control, linked to the mechanism of action of these drugs as described previously in other biological systems (Additional file 2: Table S4) [51, 52]. Since metformin is capable of causing lysosomal and mitochondrial damage, these perturbations could contribute to the enrichment of protein cargo in sEVs, such as enzymes involved in carbon and nucleotide metabolism, and autophagy and detoxification activity [53, 54]. Similarly, because albendazole disrupts the microtubule dynamic equilibrium and induces endoplasmic reticulum stress, we found down-expression of tubulins and overloading of proteasome subunit components and heat shock proteins in these sEVs [55, 56]. Among these proteins, we found enzymes that could modulate the proliferation, such as estradiol 17β-dehydrogenase (W6U9Y5, a steroidogenic enzyme that controls the last step in the formation of estrogens), which catalyzes the conversion of estrone to a more potent estrogen, 17β-estradiol (E_2_), with potential capacity for binding to parasite nuclear receptors to promote transcription [23]. In the same way, hypoxanthine-guanine phosphoribosyltransferase (W6ULW2) and GMP-synthase (W6UEZ1) are involved in purine biosynthesis, while lethal (2) giant larvae protein (W6UD39, which plays an important role in regulating cell polarity and asymmetric division) functions as tumor suppressor, controlling proliferation by Notch signaling, as has been previously reported in exosomes [57]. Moreover, we detected other enzymes such as mannose-1-phosphate guanylyltransferase-α (W6U7H3) involved in guanosine diphosphate (GDP)-mannose biosynthesis required for protein N-glycosylation, which was also identified in colon and ovarian cancer exosomes, and α-*N*-acetyl-galactosaminidase (W6UG73) and endoglycoceramidase (W6UMJ6) which remove carbohydrate residues from glycopeptides and glycolipids and are thus able to induce changes in parasite antigenicity [58, 59]. Interestingly, all these enzyme-cargo could modify the glycan moiety of parasite and/or host glycoproteins during the interaction of sEVs with their environment, which also possess highly glycosylated antigens (Additional file 3: Table S5).

Although it is well known that exosomes are naturally antigen transport carriers, this is the first work that highlights the antigenic content, including several known and potential uncharacterized antigens, of *E. granulosus* sEVs obtained in both the presence and absence of drug treatment (Fig. 2 and Additional files 3, 4: Tables S5, S6). This considerable antigenic cargo can be transferred to DCs to promote their maturation and cytokine transcription activation (Fig. 4), which allows the initiation of T-cell-mediated immune responses to improve the antiparasitic response, as has been described in other systems such as cancer cell exosomes [60]. The maturation and modulation of DCs by *E. granulosus* E/S products, including soluble antigens, affect the release of cytokines that regulate the profile of immune responses to mainly induce immune tolerance [61–63]. Similarly, a previous study demonstrated that EVs derived from this cestode exert an immunosuppressive effect on murine CD4^+^ and CD8^+^ T-cell proliferation and significantly inhibit IL-10, interferon gamma (IFN-γ), IL-6, IL-17A, and TNF-α secretion and promote IL-2 and IL-4 secretion [17]. Similarly, sEVs derived from *Taenia pisiformis* cysticerci induced M2 macrophage polarization [64]. Conversely, in the present study, the sEVs obtained from all conditions studied carried similar antigenic characteristics that allowed induction of IL-12 in DCs, favoring a Th1 response (Fig. 4) as was observed in M1-derived macrophages after exposure to exosome-like vesicles from *Schistosoma japonicum* [65]. Remarkably, the parasite sEVs obtained under metformin treatment increased the expression of pro-inflammatory cytokines such as IL-6, TNF-α, and IL-10 at even higher levels than the LPS control used in our assays (Fig. 4). In this line of evidence, a recent report showed that *E. granulosus* exosome-like vesicles induced maturation and differentiation of BMDCs towards a pro-inflammatory profile with the production of IL-6, IL-12, IL-β, TNF-α, and IFN-γ promoted by egr-miR-277a-3p and the regulation of the NF-kB p65/p50 ratio [66]. On the other hand, we also observed a decrease in the gene transcription of IL-6 and TGF-β in BMDCs exposed to control and ABZSO-derived sEVs. These data, added to the lack of IL-23 induction, corroborate results reported in murine lymphocytes, where the Th17 profile was not induced after exposure to EVs from *E. granulosus* [17]. Interestingly, EVs from *Trichinella spiralis* were reported to generate a Th1/Th2 mixed immune response characterized by the release of IL-12, IFN-γ, IL-4, and IL-10 in the serum of immunized mice [65]. Therefore, the observed heterogeneous responses are probably dependent on the composition of the EVs including the antigenic protein and other biomolecules, and especially on the context in which they were generated.

The description and characterization of sEV antigens and immunomodulatory molecules is crucial to elucidating the potential role of these vesicles as tools for the identification of new biomarkers for echinococcosis, in immunotherapy for autoimmune diseases, and for the development of a new generation of vaccines. We found several unknown putative antigens that possess a high number of epitopes (Additional file 4: Table S6). These proteins, like the known antigens present in these worm-derived sEVs (Additional file 3: Table S5), may be O-glycosylated, which may result in recognition by C-type lectins, contributing to the immunoregulatory activity of EVs [66]. Likewise, N-glycosylation could mediate vesicle internalization and consequent immunomodulation, as has been reported for *Schistosoma mansoni* EVs in monocyte-derived DCs through DC-specific ICAM-grabbing non-integrin (DC-SIGN) [67]. Therefore, extensive protein glycosylation could have a key role in the conformational integrity, antigenicity, and immunogenicity of *E. granulosus* sEV antigens. Among the proteins detected in our samples, antigen 5, which corresponds to one of the most immunogenic and abundant hydatid fluid antigens, contains at least 10 and 2 *O*- and N-glycosylation sites, respectively. Considering that antigen 5 is detected at higher concentrations in albendazole-treated patients [68], the higher abundance of this antigen in sEVs from ABZSO-treated parasites was expected, as we had previously shown such an increase for EVs obtained under treatment with loperamide [16]. However, in the present study, antigen 5 shows a similar abundance in all studied conditions, indicating that the higher ratio observed in albendazole-treated patients probably corresponds to an increase in cyst permeability rather than greater antigen production and release in either a soluble or sEV-linked manner. As we mentioned before, the increase in cyst permeability by albendazole favors the leakage of antigens that promote antibody production [69] and the pro-inflammatory response, which is beneficial for limiting parasitic progression in both CE and alveolar echinococcosis [25, 70, 71].

Moreover, we also identified several proteins associated with immunomodulation and interaction with the host (Fig. 3 and Additional file 1: Fig. S1). A well-represented protein was the T-cell immunomodulatory protein (W6V8B8 ortholog of Em-TIP), which has previously been characterized as an E/S product in* E. multilocularis* primary cell cultures involved in metacestode development and in the promotion of IFN-γ release by murine Th1 cells during the early stages of infection [72]. In addition, potential orthologs of this protein were reportedly involved in the regulation of inflammatory cytokines and in an acute graft-versus-host disease model, in *Plasmodium berghei* infection, and in *Cryptosporidium parvum* invasion [73–76]. Therefore, considering their adhesion and immunomodulatory characteristics, the Eg-TIP present in sEVs could have a role in parasite establishment and in the modulation of the early Th1 response. The LTA4 hydrolase (W6UMY4) and prostaglandin-E_2_ 9-ketoreductase (W6U2E2) detected in our samples could also be involved in the pro-inflammatory profile that generates the *E. granulosus* sEVs. In this sense, these enzymes mediate the synthesis of pro-inflammatory mediators that represent key factors in type 2 inflammation [77, 78] which, among other functions, can induce granulocyte recruitment as has been reported in *Caenorhabditis elegans* and *Nippostrongylus brasiliensis* [79].

Additionally, in this study, we detected several proteins related to protein–ligand or protein–protein interactions such as LRR proteins (W6UG49 and W6UFQ5, Fig. 3 and Additional file 5: Fig. S1). As an evolutionary conserved strategy, plants, invertebrates, and vertebrates, use LRR-containing domains to sense pathogen patterns as a first line of defense [80]. Parasite LRR proteins could play a critical role in mimicking and desensitizing host sensors [81, 82]. Moreover, in pathogens such as *Leishmania* and *Leptospira interrogans*, proteins with LRR domains have been shown to be involved in mediating pathogenicity, host cell attachment, and invasion [83, 84]. On the other hand, we also identified a scavenger receptor class B member (W6V978) homolog to a transmembrane glycoprotein found in macrophages, microglia, microvascular endothelium, cardiac and skeletal muscle, adipocytes, and platelets implicated in angiogenesis, atherosclerosis, phagocytosis, inflammation, lipid metabolism, and removal of apoptotic cells [85]. The expression of a CD36-like class B scavenger receptor in *S. mansoni* and *Opisthorchis viverrini* has been associated with the acquisition of host lipids (low-density lipoprotein [LDL], intermediate-density lipoprotein [IDL], and fatty acids) probably for nutritional, developmental, and/or immune evasion purposes [86, 87]. Therefore, the presence of this homologous protein in the *E. granulosus* sEVs could be associated with the amelioration in lipid uptake in the target organ for improving parasite growth. Finally, two basement membrane-specific heparan sulfate proteoglycan core proteins (W6V2K4 and W6UKD6) were detected in *Echinococcus* sEVs, which have also been found in E/S products of *Hymenolepis diminuta* and associated with transport [88]. Interestingly, in the same line of evidence as our results, homologs for these proteins present in mast cell-derived EVs may be associated with inactive TGF-β1, which mediates signaling in endosomes of the recipient cell, allowing regulation of its phenotype and function [89].

Overall, this work revealed that sEVs obtained from *E. granulosus* larvae contributed to the process of parasite–host communication and to the initial type 1 immune response in the host, which was enhanced by drug treatment with ABZSO and metformin because both drugs tend to increase IL-12 [24, 25, 90]. We determined that the sEVs of this cestode induced the production of pro-inflammatory cytokines from BMDC, which promoted a Th1 profile in T cells, mainly with those vesicles derived from metformin treatment. These results contribute to our understanding of the high pharmacological efficacy of these drugs in in vivo echinococcosis experimental models, where the sEVs can participate in the potentiation of the host immune response for parasite growth control [31, 91]. In this study, we provide the first evidence of the common, enriched, and unique protein cargo of sEVs obtained under albendazole and metformin treatment. Given that these sEVs have potential immunomodulatory functions, future studies may be performed to identify other macromolecule components such as carbohydrates, lipids, and small RNAs capable of altering the host immune system.

## Supplementary Information

**Additional file 1: Tables S1**–**S3.** Proteomic analysis of extracellular vesicles from control and metformin- or ABZSO-treated protoscoleces of *Echinococcus granulosus*.

**Additional file 2: Table S4.** Differential enrichment of the common proteins identified in sEVs released under metformin- or ABZSO-treated protoscoleces of *Echinococcus granulosus* with respect to the control.

**Additional file 3: Table S5. **Antigenic cargo of *Echinococcus granulosus* extracellular vesicles obtained from control and drug-treated parasites.

**Additional file 4: Table S6. **Uncharacterized proteins identified in *Echinococcus granulosus *extracellular vesicles.

**Additional file 5: Figure S1. **Structural organization of the immune modulator proteins identified in sEVs from *E. granulosus* protoscoleces based on the functional domains. Conserved domains were assigned using the CDD, CDART, and SWISS-MODEL server. Protein identity and Uniprot ID are indicated in the left column. Protein length is indicated from the N-terminal to the C-terminal end. The right column indicates the identity percentages and the GMQE (global model quality estimate) of each immunomodulatory protein with different queries obtained from the SWISS-MODEL server. The SWISS-MODEL template ID for each query is indicated in the corresponding row. Leucine-rich repeat (LRR)-containing protein (W6UG49) contains a domain with LRRs that corresponds to the ribonuclease inhibitor-like subfamily (pink bar) between 220 and 489 amino acids (aa). T-cell immunomodulatory protein (W6V8B8) contains an N-terminal Rab- GTPase-TBC domain in the first 260 aa, which belongs to the RabGAP-TBC superfamily (cl02495, orange bar), two transmembrane domains (TM, black bars) including 256–278 and 806–828 aa, and a domain corresponding to a bifunctional aldos-2-ulose dehydratase including 347–710 aa (yellow bar). The full length of profilin (U6JIW0) corresponds to the PROF superfamily (cl00123, red bar). Basigin (W6U7D3) contains an immunoglobulin (Ig) domain found in the Ig superfamily (cl11960, light green bar) between 140 and 235 aa, a TM domain including 239–262 aa (black bar) and the full length shows structural identity with basigin orthologs (dark green bar). B-cell receptor-associated protein (W6U5T3) contains a structural maintenance of chromosomes (Smc) superfamily domain between 22 and 150 aa (cl34174, blue bar), six TM domains including 514–536, 567–589, 604–626, 811–833, 853–875, and 913–935 aa (black bars), and two B-cell receptor-associated protein domains including the Bap31 superfamily (cl02219) and Bap31 Bap29 C superfamily (cl39451) including 809–947 and 989–1053 aa, respectively (light pink bars). Ras C3 botulinum toxin substrate 2 (U6JJ69) contains a P-loop NTPase superfamily domain between 3 and 176 aa (cl38936, white bar) and the full length shows structural identity with Ras-related c3 botulinum toxin substrate 2 and cell division control protein 42 homolog (Cdc42) orthologs (light blue bar). Leukotriene A-4 hydrolase (W6UMY4) contains a leukotriene A-4 hydrolase/aminopeptidase domain between 32 and 635 aa (TIGR02411, brown bar) and the full length shows structural identity with leukotriene A-4 hydrolase orthologs (purple bar). N(G),N(G)-dimethylarginine dimethylaminohydrolase (W6V8P5) contains an amidinotransferase superfamily domain between 4 and 149 aa (cl19186, light orange bar) and the full length shows structural identity with dimethylargininase orthologs (dark orange bar). Prostaglandin-E_2_ 9-reductase (W6U2E2) contains an aldo-keto reductase (AKR) superfamily domain including 15–277 aa (cl00470, blue bar) and the full length shows structural identity with prostaglandin-E_2_ 9-reductase orthologs (violet bar). Maspardin (W6U7Y4) contains an abhydrolase 6 superfamily domain between 62 and 270 aa (cl38364, light green bar) and the full length shows structural identity with maspardin orthologs (dark green bar). LRR-containing protein 57 (W6UFQ5) contains a domain with LRRs which corresponds to the inl-like NEAT 1 superfamily (cl41270, pink bar) between 7 and 217 aa. Scavenger receptor class B member (W6V978) contains two TM domains including 12–34 and 467–489 aa (black bars), a CD36 superfamily domain including 35–479 aa (cl10574, orange bar), and the full length shows structural identity with Scavenger receptor class B orthologs (purple bar). Basement membrane-specific heparan sulfate proteoglycan core protein (W6V2K4) consists of a TM domain between 13 and 35 aa (black bar); six immunoglobulin-like domains including 152–227, 850–948, 1737–1836, 2701–2777, 2815–2898, and 7746–7841 aa (green bars); a low-density lipoprotein receptor domain class A between 723 and 803 (blue bar); two laminin B domains including 1049–1185 and 1330–1374 aa (yellow bars) and a TNF receptor superfamily domain between 1198 and 1309 aa (red bar). Basement membrane-specific heparan sulfate proteoglycan core protein (W6UKD6) consists of a TM domain between 6 and 24 aa (black bar); three immunoglobulin-like domains including 233–314, 328–402, and 745–831 aa (green bars) and three low-density lipoprotein receptor domains including 617–649, 679–697, and 705–740 (blue bars).

## Data Availability

The data described in this article are presented in the main manuscript and in the additional supporting files.

## Abbreviations

ABZSO: Albendazole sulfoxide
BMDCs: Bone marrow-derived dendritic cells
CE: Cystic echinococcosis
DCs: Dendritic cells
DLS: Dynamic light scattering
E/S: Excretory/secretory
EVs: Extracellular vesicles
FACS: Fluorescence-activated cell sorting
LPS: Lipopolysaccharide
PBS: Phosphate-buffered saline
SDS-PAGE: Sodium dodecyl sulfate–polyacrylamide gel electrophoresis
sEVs: Small extracellular vesicles
TEM: Transmission electron microscopy
