# Supplementary material for: Characterization of protein cargo of Echinococcus granulosus extracellular vesicles in drug response and its influence on immune response
Source: Parasit Vectors. 2023 Jul 29;16:255. doi: 10.1186/s13071-023-05854-6 (PMC10387209; doi:10.1186/s13071-023-05854-6)
Supplement: Supplementary file 2 — Additional file 2: Table S4. Differential enrichment of the common proteins identified in sEVs released under metformin- or ABZSO-treated protoscoleces of Echinococcus granulosus with respect to the control. [file 13071_2023_5854_MOESM2_ESM.docx]

**Additional file 2: Table S4.** Differential enrichment of the common proteins identified in sEVs released under metformin or ABZSO-treated protoscoleces of *Echinococcus granulosus* respect to the control.

| **Fold change*: ABZSO versus Control** | | | |
| --- | --- | --- | --- |
| **Up-regulated proteins** | | **Down-regulated proteins** | |
| (U6J1G7) 26S proteasome non ATPase regulatory subunit 8 | 2 | (W6UDI6) Tubulin alpha-1C chain | -1.3 |
| (W6UD45) 26S proteasome non-ATPase regulatory subunit | 1.5 | (W6UJD0) Tubulin beta-3 chain | -1.15 |
| (W6U6K8) 26S proteasome non-ATPase regulatory subunit 3 | 0.3 | (W6UST3) Tubulin alpha chain | -1.22 |
| (W6UBD1) Heat shock cognate protein | 3 | (W6V3X7) Tubulin beta-2 chain | -1.1 |
| (W6UGS9) Heat shock cognate protein | 1.3 | (W6US08) Tubulin beta chain | -1.6 |
| (W6UM50) Heat shock protein HSP 90-alpha | 1.15 | (U6J5Z8) 14-3-3 protein zeta | -1.1 |
|  |  | (W6UE99) Phosphoglycerate kinase | -1.1 |
|  |  | (W6UZC4) Phosphoglucomutase | -2 |
| **Fold change*: Metformin versus Control** | | | |
| (W6UGY6) Pyruvate kinase isozyme R | 2 | (W6V2K4) Basement membrane-specific heparan sulfate proteoglycan core protein | -3 |
| (W6UI91) Pyruvate kinase isozymes R/L | 2 | (W6UKD6) Basement membrane-specific heparan sulfate proteoglycan core protein | -2 |
| (W6UM60) Pyruvate kinase | 2 |  |  |
| (W6UAI6) Malate dehydrogenase | 0.67 |  |  |
| (W6V798) Glucose-6-phosphate isomerase | 0.43 |  |  |
| (W6UVI3)L-lactate dehydrogenase | 0.5 |  |  |
| (W6UHV2) Phosphoenolpyruvate carboxykinase [GTP] | 0.19 |  |  |
| (W6V1T8) Glyceraldehyde-3-phosphate dehydrogenase | 0.08 |  |  |
| (Q56J98) 14-3-3 protein | 0.27 |  |  |
| (U6JGI4) 14-3-3 protein beta:alpha | 0.06 |  |  |
| (U6JEE0) 14-3-3 protein epsilon | 0.3 |  |  |
| (W6UCC3) Glutathione peroxidase | 0.5 |  |  |
| (U6JFS3) Glutathione S transferase | 0.22 |  |  |
| (W6U7N2) Lysosomal acid lipase/cholesteryl ester hydrolase | 2 |  |  |
| (W6UBQ8) Lysosomal aspartic protease | 0.33 |  |  |
| (W6UQX7) Guanine nucleotide-binding protein G(S) sub. α | 5 |  |  |
| (W6UU29) Guanine nucleotide-binding protein G(Q) sub. α | 1 |  |  |
| (W6UAF9) Guanine nucleotide-binding protein G(O) sub. β | 0.66 |  |  |
| (W6UI48) Guanine nucleotide-binding protein | 0,5 |  |  |
| (W6UBY3) Guanine nucleotide-binding protein G(O) sub. α | 0.25 |  |  |
| (U6J2Y8) Guanine nucleotide binding protein sub. β | 0.25 |  |  |
| (W6ULW2) Hypoxanthine-guanine phosphoribosyltransferase | 2 |  |  |
| (W6UYD3) T-complex protein 1 subunit gamma | 0.4 |  |  |
| (W6V2J0) T-complex protein 1 subunit eta | 0.25 |  |  |
| (W6UT85) T-complex protein 1 subunit delta | 0.11 |  |  |

* Fold change was calculated by the following equation: (Y - X)/X where “Y” corresponds to the number of peptides identified in sEVs from the treated sample (metformin or ABZSO) and “X” corresponds to the number of peptides from control sEVs.
