# Supplementary material for: Characterization of protein cargo of Echinococcus granulosus extracellular vesicles in drug response and its influence on immune response
Source: Parasit Vectors. 2023 Jul 29;16:255. doi: 10.1186/s13071-023-05854-6 (PMC10387209; doi:10.1186/s13071-023-05854-6)
Supplement: Supplementary file 3 — Additional file 3: Table S5. Antigenic cargo of Echinococcus granulosus extracellular vesicles obtained from control and drug-treated parasites. [file 13071_2023_5854_MOESM3_ESM.docx]

**Additional file 3. Table S5.** Antigenic cargo of *Echinococcus granulosus* extracellular vesicles obtained from control and drug-treated parasites.

| **Uniprot ID** | **Identified protein** | **Protein length (aa)** | **Peptides**  **C/M/A** | **Epitopes**  **above 0.6 threshold with ≥15 aa** | **O-glycoslilation**  **Prediction**  **(>0,5-0.75-1)** | **N-glycosilation Prediction**  **(>0,5-0.75-1)** |
| --- | --- | --- | --- | --- | --- | --- |
| U6JGI4 | 14-3-3 protein beta:alpha OS=Echinococcus granulosus GN=EGR_10354 | 248 | 17/18/18 | - | **5, 76, 77** | **185, 236** |
| Q56J98 | 14-3-3 protein OS=Echinococcus granulosus GN=EGR_03128 | 247 | 15/19/17 | - | **3,68,233,238** | **176, 227** |
| U6JDG7 | Tegumental protein OS=Echinococcus granulosus GN=EGR_05835 | 180 | 16/15/12 | 1 | **73,88,95,98** | **-** |
| W6V2P2 | Hydatid disease diagnostic antigen P-29 OS=Echinococcus granulosus GN=P29 | 238 | 15/14/15 | - | **14,27,28,34,171** | **127, 139** |
| U6JBW8 | Major egg antigen p40 OS=Echinococcus granulosus GN=EGR_03078 | 314 | 14/16/14 | 1 | **14,198, 208, 218** | **273** |
| W6UXT1 | Major egg antigen OS=Echinococcus granulosus GN=EGR_01883 | 315 | 13/15/15 | 2 | **11, 21, 22, 210,212, 214, 215, 332** | **148, 162, 163** |
| U6JEE0 | 14-3-3 protein epsilon OS=Echinococcus granulosus GN=EGR_01834 | 255 | 10/13/12 | - | **3, 5, 160, 237, 248** | **180, 231** |
| W6U648 | Tegumental protein OS=Echinococcus granulosus GN=EGR_08521 | 99 | 9/9/8 | - | **36** | **-** |
| W6UDE2 | Endophilin-B1 OS=Echinococcus granulosus GN=EGR_05824 | 274 | 6/9/8 | - | **12, 19, 183, 254, 259, 163** | **-** |
| W6UFE0 | Sj-Ts4 protein OS=Echinococcus granulosus GN=EGR_08263 | 306 | 7/7/7 | 1 | **58, 255, 258, 261, 262** | **210, 220** |
| W6UE73 | Antigen EG13 OS=Echinococcus granulosus GN=EGR_05904 | 634 | 7/7/7 | 6 | **78,182, 311,323, 329, 330,334,336, 337, 340, 342, 343, 353, 354, 356, 358, 361, 362, 363, 367, 368, 377, 378, 384, 386, 390, 394, 395, 411, 412, 415, 425, 433, 435, 436, 439, 459, 465, 475, 480, 481, 490, 569, 576, 582, 590, 596, 598, 599, 600, 605, 607, 611, 616, 617** | **268, 423, 426** |
| W6UGD8 | Endophilin-A1 OS=Echinococcus granulosus GN=EGR_05034 | 321 | 5/7/6 | 1 | **38, 104, 143, 147, 156, 158, 161, 197, 205, 209, 221, 222, 225, 226, 231, 232, 239, 250, 252, 253, 261, 262** | **-** |
| W6U646 | Tegumental protein OS=Echinococcus granulosus GN=EGR_08411 | 428 | -/2/2 | 4 | **76, 221, 222, 226, 228, 230, 236, 240, 241, 243, 244, 246, 247, 248, 249, 259, 260, 261, 262, 263, 264, 265, 287, 289, 293, 295, 302, 308, 320, 322, 325, 326, 329, 330, 332, 333, 335** | **318** |
| W6URS7 | Major egg antigen OS=Echinococcus granulosus GN=EGR_01882 | 503 | 3/5/2 | 5 | **57, 60, 62, 63, 66, 70, 72, 73, 75, 76, 78, 81, 83, 85, 86, 177, 179, 191, 192, 195, 196, 197, 198, 209, 210, 212, 213, 214, 215, 218, 221, 226, 229, 231, 233, 234, 235, 236, 237, 252, 315, 323, 383, 384, 391, 394, 395, 396, 397, 401, 404, 451** | **-** |
| U6J5Z8 | 14-3-3 protein zeta OS=Echinococcus granulosus GN=EGR_03190 | 256 | 9/6/8 | - | **6, 7, 45, 73, 79, 84, 244** | **187** |
| I1WXU1 | Ag5 OS=Echinococcus granulosus GN=EGR_04862 | 484 | 3/4/3 | 4 | **76, 101, 103, 128, 131, 146, 151, 155, 180, 373** | **55, 213** |
| U6IZE6 | Cupin 2 barrel domain containing protein OS=EGR_02662 | 105 | 2/3/2 | - | ---------- | **-** |
| U6JGV2 | Tegument antigen OS=Echinococcus granulosus GN=EGR_09689 | 115 | 3/3/2 | 1 | **8, 9** | **16** |
| W6UEY0 | Tegument antigen OS=Echinococcus granulosus GN=EGR_08410 | 234 | -/2/- | 1 | **48, 50, 116, 130, 131, 135** | **-** |
| W6U6B4 | Tegument antigen OS=Echinococcus granulosus GN=EGR_08443 | 168 | 2/-/- | 1 | **75, 77** | **136** |
