# Supplementary material for: Characterization of protein cargo of Echinococcus granulosus extracellular vesicles in drug response and its influence on immune response
Source: Parasit Vectors. 2023 Jul 29;16:255. doi: 10.1186/s13071-023-05854-6 (PMC10387209; doi:10.1186/s13071-023-05854-6)
Supplement: Supplementary file 4 — Additional file 4: Table S6. Uncharacterized proteins identified in Echinococcus granulosus extracellular vesicles. [file 13071_2023_5854_MOESM4_ESM.docx]

**Additional file 4. Table S6.** Uncharacterized proteins identified in *Echinococcus granulosus* extracellular vesicles**.**

| **Uniprot ID** | **Peptides**  **C/M/A** | **Protein length (aa)** | **Sec-P**  **Score**  **(>0.6)/ Signal P** | **TM domain**  **(HMMTOP, TMHMM, DAS)**  **(position)** | **Epitopes**  **above 0.6 threshold with ≥15 aa** |
| --- | --- | --- | --- | --- | --- |
| W6UCJ7 EGR_09161 | 12/18/14 | 2534 | 0.230/- | - | 31 |
| W6TZB8 EGR_11068 | 9/13/7 | 2010 | 0.372/- | - | 10 |
| W6U118 EGR_10334 | 6/6/6 | 274 | 0.703/+ | - | - |
| W6UR49 EGR_00739 | 10/7/9 | 414 | 0.264/- | - | 1 |
| W6V5G4 EGR_03588 | 4/7/4 | 1190 | 0.437/- | - | 3 |
| W6U9T7 EGR_09890 | 3/7/6 | 668 | 0.433/- | 1 (493-503) | 5 |
| W6UIK2 EGR_04149 | 2/4/2 | 289 | 0.292/- | - | 2 |
| W6UX40 EGR_02181 | 3/7/5 | 709 | 0.450/- | - | 2 |
| W6UXT9 EGR_01893 | -/6/3 | 293 | *0.761/- | - | - |
| W6UT44 EGR_03591 | -/2/- | 1216 | 0.512/- | - | 11 |
| W6UL74 EGR_06281 | 3/3/3 | 398 | *0.733/- | - | 1 |
| W6UQM2 EGR_01113 | -/2/- | 85 | *0.889/- | - | - |
| W6ULU3 EGR_02967 | -/3/2 | 298 | 0.544/- | - | 2 |
| W6VCD1  EGR_00474 | 2/3/2 | 682 | *0.778/- |  | - |
| W6U3U1  EGR_09889 | -/2/2 | 331 | *0.734/- | - | 1 |
| W6UD40  EGR_05958 | -/2/- | 349 | 0.596/- | 5 (26-34 ; 89-111 ; 132-153; 181-201; 298-319) | - |
| U6J5W7  EGR_02661 | 2/2/2 | 105 | 0.472/- | - | - |
| W6V0C1  EGR_05680 | -/2/- | 369 | 0.338/- | 1 (94-111) | 2 |
| W6UCJ2  EGR_06189 | -/2//2 | 259 | 0.496/- | - | - |
| W6U1R1  EGR_10690 | 2/2/- | 167 | 0.520/- | - | - |
| W6VAV4  EGR_01042 | 2/2/- | 3445 | 0.270/- | - | 4 |
| W6UIJ6 EGR_04177 | 4/6/5 | 155 | 0.324/- | - | - |
| W6UTT6 EGR_00309 | 2/-/2 | 164 | 0.537/- | 1 (38-57) | - |
| W6UXX5 EGR_01519 | 2/-/3 | 380 | 0.534/- | - | 2 |
| U6JME9 EGR_07945 | 3/-/3 | 1018 | 0.489/+ | 1 (891-908) | 4 |
| W6U2P3 EGR_09757 | 2/-/2 | 192 | *0.727/- | - | 1 |
| W6ULI1 EGR_06176 | 4/-/- | 2582 | 0.552/- | 13 (58-77; 96-109; 141-149; 174-191; 202-215; 255-256; 288-310; 363-372; 411-422; 440-453; 474-493; 506-520; 1383-1401) | 11 |
| W6VC79 EGR_00993 | -/-/4 | 385 | *0.657/- | - | 1 |
| W6U780  EGR_08893 | 11/6/10 | 1019 | *0.745/- | 5 (151-171; 313-334; 597-617; 656-677; 986-1005) | 1 |
| W6ULE5  EGR_03176 | 25/19/22 | 595 | 0.299/- | - | 5 |
| W6U6P0  EGR_08255 | 3/-/3 | 344 | 0.585/+ | 1 (6-23) | - |
| W6VBX7  EGR_00893 | 8/11/8 | 415 | 0.563/- | - | - |
| W6UX03  EGR_07135 | 14/2/14 | 1482 | 0.182/- | 2 (579-594; 1342-1363) | 3 |
| W6U1N0  EGR_10196 | 7/5/5 | 293 | *0.924/- | 3 (11-32; 72-93; 102-123) | 2 |
| W6UNQ7  EGR_01786 | 19/19/15 | 1291 | 0.097/- | - | 15 |
| W6UJ45  EGR_06921 | 3/-/3 | 187 | 0.717/+ | 1 (6-24) | - |
| W6U6B2  EGR_11266 | 2/-/2 | 121 | *0.740/- | - | - |
| W6UN14  EGR_02580 | 4/5/3 | 269 | *0.906/- | 1 (68-86) | - |
| W6UCY9  EGR_05903 | 10/10/9 | 245 | 0.379/- | - | - |
| W6U828  EGR_07846 | 7/8/9 | 108 | *0.774/- | - | - |
| W6U8U0  EGR_08238 | 4/5/4 | 316 | 0.776/+ | - | - |
| W6UIJ6  EGR_04177 | 4/6/5 | 155 | 0.324/- | - | - |
| W6U9F5  EGR_10026 | 3/-/- | 374 | 0.380/- | 1 (42-66) | 1 |
| W6USF9  EGR_01663 | -/2/2 | 198 | *0.616/- |  | - |
| U6JPQ6  EGR_07941 | 4/4/3 | 439 | 0.221  /- | - | 4 |
| U6IV22  EGR_00080 | 0/4/2 | 225 | 0.875  /- | - | 2 |
| W6UK94  EGR_03547 | 8/13/10 | 457 | *0.681/- | - | - |
| W6UE48  EGR_05999 | 5/5/5 | 383 | 0.245/- | - | 3 |
| W6UCI3 EGR_09176 | 2/3/2 | 247 | 0.801/+ | 2 (21-31; 191-206) | - |
| W6UL07 EGR_02967 | 2/4/2 | 418 | *0.621/- | 1 (28-36) | 1 |
| W6UD40 EGR_05958 | -/2/- | 349 | 0.596/- | 5 (26-34; 89-111;  132-153; 181-201; 297-319) | - |
| W6UR20 EGR_01945 | 3/3/3 | 441 | 0.491/- | - | - |
| W6UXL9 EGR_01793 | -/2/- | 124 | 0.512/+ | 1 (15-35) | - |
| W6VD71 EGR_00168 | -/2/2 | 171 | *0.922/- | 2 (7-25; 71-81) | 1 |
| W6UQP1  EGR_09402 | 3/2/2 | 269 | *0.908/- | 1 (26-40) | - |
| U6J0J5  EGR_01398 | -/2/- | 306 | 0.483/- | - | 2 |
| W6UDZ7 EGR_06063 | 3/-/4 | 775 | 0.375/- | 1 (255-271) | 2 |
| W6UAY5 EGR_06627 | 2/-/- | 258 | *0.833/- | - | 3 |
| W6UJW8 EGR_03721 | 3/-/- | 798 | 0.242/- | - | 6 |
| W6UE44  EGR_05994 | 11/9/9 | 388 | 0.303/- | - | 4 |
| W6UY44  EGR_01609 | 7/10/5 | 465 | 0.505/- | - | 7 |
| W6UFB6  EGR_05274 | 4/4/5 | 258 | 0.377/- | 5 (26-40; 63-81; 102-122; 160-178; 203-219) | - |
| W6U7G9  EGR_08851 | 2/3/- | 234 | 0.380/- | - | 2 |
| U6JMR8  EGR_08855 | 4/6/6 | 207 | 0.374/- | - | 2 |
| U6IWW9  EGR_01669 | 2/-/- | 321 | 0.316/- | - | 2 |
| U6IYX9  EGR_01671 | 5/5/4 | 647 | 0.386/- | - | 6 |
| U6J7X5  EGR_01858 | -/2/- | 241 | *0.643/- | 4 (29-46, 56-74, 83-102, 113-128) | 1 |
| U6J321  EGR_02048 | -/2/- | 422 | 0.432/- | - | 3 |
| U6IWR1  EGR_01670 | -/3/2 | 444 | *0.754/- | - | 3 |
| W6URV7  EGR_01508 | 3/4/3 | 612 | 0.614/+ | 3 (171-189, 216-234, 544-563) | - |

SecretomeP: Secretion prediction according to SecretomeP 2.0 server. Protein sequences were analyzed in terms of mammalian secretion pathways (>0.6). Numbers correspond to NN-score.

SignalP: Secretion prediction according to Signal 4.0 server. +/- indicates presence/absence of signal peptide, respectively.

* indicates protein secretion by non-classical pathway
